# The genetic landscape of the human solute carrier (SLC) transporter superfamily

**DOI:** 10.1007/s00439-019-02081-x

**Published:** 2019-11-02

**Authors:** Lena Schaller, Volker M. Lauschke

**Affiliations:** Department of Physiology and Pharmacology, Section of Pharmacogenetics, Karolinska Institutet, 171 77 Stockholm, Sweden

## Abstract

The human solute carrier (*SLC*) superfamily of transporters is comprised of over 400 membrane-bound proteins, and plays essential roles in a multitude of physiological and pharmacological processes. In addition, perturbation of SLC transporter function underlies numerous human diseases, which renders SLC transporters attractive drug targets. Common genetic polymorphisms in *SLC* genes have been associated with inter-individual differences in drug efficacy and toxicity. However, despite their tremendous clinical relevance, epidemiological data of these variants are mostly derived from heterogeneous cohorts of small sample size and the genetic *SLC* landscape beyond these common variants has not been comprehensively assessed. In this study, we analyzed Next-Generation Sequencing data from 141,456 individuals from seven major human populations to evaluate genetic variability, its functional consequences, and ethnogeographic patterns across the entire *SLC* superfamily of transporters. Importantly, of the 204,287 exonic single-nucleotide variants (SNVs) which we identified, 99.8% were present in less than 1% of analyzed alleles. Comprehensive computational analyses using 13 partially orthogonal algorithms that predict the functional impact of genetic variations based on sequence information, evolutionary conservation, structural considerations, and functional genomics data revealed that each individual genome harbors 29.7 variants with putative functional effects, of which rare variants account for 18%. Inter-ethnic variability was found to be extensive, and 83% of deleterious SLC variants were only identified in a single population. Interestingly, population-specific carrier frequencies of loss-of-function variants in *SLC* genes associated with recessive Mendelian disease recapitulated the ethnogeographic variation of the corresponding disorders, including cystinuria in Jewish individuals, type II citrullinemia in East Asians, and lysinuric protein intolerance in Finns, thus providing a powerful resource for clinical geneticists to inform about population-specific prevalence and allelic composition of Mendelian *SLC* diseases. In summary, we present the most comprehensive data set of *SLC* variability published to date, which can provide insights into inter-individual differences in SLC transporter function and guide the optimization of population-specific genotyping strategies in the bourgeoning fields of personalized medicine and precision public health.

**Electronic supplementary material:**

The online version of this article (10.1007/s00439-019-02081-x) contains supplementary material, which is available to authorized users.

## Introduction

The solute carrier (*SLC*) gene superfamily is one of two major human gene families encoding transporters of endogenous and exogenous compounds. *SLC*s constitute the second-largest family of membrane proteins in the human genome with over 400 proteins classified into 65 subfamilies based on sequence similarity (Fredriksson et al. 2008; Höglund et al. 2011; Schlessinger et al. 2013). Substrate specificity differs substantially across the various subfamilies. While some subfamilies, such as the carbohydrate and long chain fatty acid transporters of the *SLC2* and *SLC27* subfamilies, transport only few physicochemically homogenous substrates (Anderson and Stahl 2013; Mueckler and Thorens 2013), transporters of the *SLC22* family mediate the translocation of various dissimilar ions, including organic cations, anions, and zwitterions (Koepsell 2013). Most SLC transporters are equilibrative, making use of electrochemical and concentration gradients to facilitate the uptake of their substrates into cells. Transport mechanisms can differ within subfamilies, however, as seen in the secondary active symporters and antiporters of the *SLC4* bicarbonate transporter family (Romero et al. 2013).

Due to their essential roles in the transport of a plethora of essential organic and inorganic substrates and the high number (> 100) of SLC transporters that have been associated with human genetic disorders, SLC transporters are being increasingly investigated as potential drug targets. One prominent example is the development of blockbuster SGLT2 (encoded by *SLC5A2*) inhibitors for the treatment of diabetic hyperglycaemia, which was inspired by associations between *SLC5A2* mutations and familial renal glucosuria (OMIM identifier 233100). Besides their role as drug targets, SLC transporters play fundamental roles in the disposition of numerous drugs, including various chemotherapeutics, antidiabetics, and diuretics. Given the extensive genomic coverage and the critical role that SLC transporters play in mediating drug pharmacokinetics and -dynamics (PK/PD), the genetic variability of *SLC* genes is of considerable interest for human genetics, as well as for drug discovery and development programs.

In the last decade, seminal studies have contributed substantially to our understanding of the link between *SLC* variability and drug response. Prominent examples include the association between variants in *SLC22A1*, encoding OCT1, and pharmacokinetics and response to metformin (Dujic et al. 2015; Sundelin et al. 2017) and variations in *SLC19A1*, encoding the reduced folate transporter RFT, with toxicity of antifolate metabolites (Bohanec Grabar et al. 2012; Corrigan et al. 2014; Lima et al. 2014). However, these studies were only powered to detect associations with common variations. Importantly, recent population-scale sequencing projects revealed that rare variations with minor allele frequencies (MAF) < 1% greatly outnumber common variants in genes involved in drug absorption, distribution, metabolism, and excretion (ADME) (Bush et al. 2016; Kozyra et al. 2017; Wright et al. 2018; Zhou and Lauschke 2018). Rare variations are enriched in variants with functional consequences and commonly have increased effect sizes compared to common variants when analyzed in relation to disease (Ingelman-Sundberg et al. 2018; Manolio et al. 2009). While the extent and functional importance of rare variants is becoming increasingly appreciated, SLC transporters are understudied (César-Razquin et al. 2015) and their genetic landscape remains to be systematically analyzed.

Here, we systematically mapped the genetic variability of the human *SLC* transporter superfamily by analyzing consolidated whole-exome and whole-genome sequencing data (WES and WGS, respectively) from 141,456 individuals across seven major populations. We profiled the *SLC* genetic variability, its functional consequences, and ethnogeographic distribution using 13 partly orthogonal computational predictors, as well as structural mapping approaches using experimental high-resolution crystal structures. The obtained data set constitutes the most comprehensive analysis of genetic *SLC* variability published to date and provides valuable insights into inter-individual and inter-ethnic differences in transporter function with important implications for drug disposition, efficacy, and toxicity, as well as population-specific prevalence of Mendelian SLC diseases.

## Materials and methods

### Data collection and annotation

Genetic variability data of 401 genes comprising the human *SLC* superfamily were collected from the Genome Aggregation Database (gnomAD) version 2.1 (Lek et al. 2016). The use of these data did not require separate ethical approval, as the data are released under the Fort Lauderdale Agreement. In total, we analyzed sequencing data of 141,456 unrelated individuals spanning seven worldwide populations (64,603 Non-Finnish Europeans, 12,562 Finns, 12,487 Africans, 9977 East Asians, 15,308 South Asians, 17,720 Latinos, 5185 Ashkenazi Jews, and 3614 from other populations). Variants with low confidence calls were removed. Rare and common genetic SNVs were defined as variants with MAF < 1% and MAF ≥ 1%, respectively. Copy-number variants’ (CNVs) data from 59,451 individuals were obtained from the Exome Aggregation Consortium and analyzed as previously described (Santos et al. 2018). Linkage analysis was performed using LDLink (Machiela and Chanock 2015). Disease associations for the relevant *SLC* genes were obtained from the Online Mendelian Inheritance in Man (OMIM) database. Deleterious variants in disease-associated genes were filtered for benign variants using ClinVar Miner (Henrie et al. 2018).

### Computational functionality predictions

Missense variants were analyzed using an array of partly orthogonal algorithms that predict the functional impact of genetic variations based on sequence information, evolutionary conservation, structural considerations, and functional genomics data. Specifically, we used SIFT (Ng and Henikoff 2001), Polyphen-2 (Adzhubei et al. 2010), Likelihood Ratio Tests (Chun and Fay 2009), MutationAssessor (Reva et al. 2011), FATHMM (Shihab et al. 2012), PROVEAN (Choi et al. 2012), VEST3 (Carter et al. 2013), CADD (Kircher et al. 2014), DANN (Quang et al. 2015), FATHMM-mkl (Shihab et al. 2015), MetaSVM (Dong et al. 2015), MetaLR (Dong et al. 2015), and GERP++ (Davydov et al. 2010). We considered all variants that resulted in the gain of a stop codon, the loss of the start codon, that caused frameshifts or that disrupted canonical splice sites as loss-of-function variants.

### Structural modeling

The secondary structures of GLUT1 (*SLC2A1*) and ENT1 (*SLC29A1*) were obtained from UniProt (UniProt IDs 4PYP and 6OB6, respectively). The structure of OCT1 (*SLC22A1*) was predicted using Phyre2 (Kelley et al. 2015) as no high-resolution crystal structure for this transporter was available. Confidence and coverage scores were ≥ 100% and 80%, respectively. Structures were modeled using PyMOL version 2.3.

## Results

### Overview of the genetic variability of the human *SLC* superfamily

**Fig. 1:**
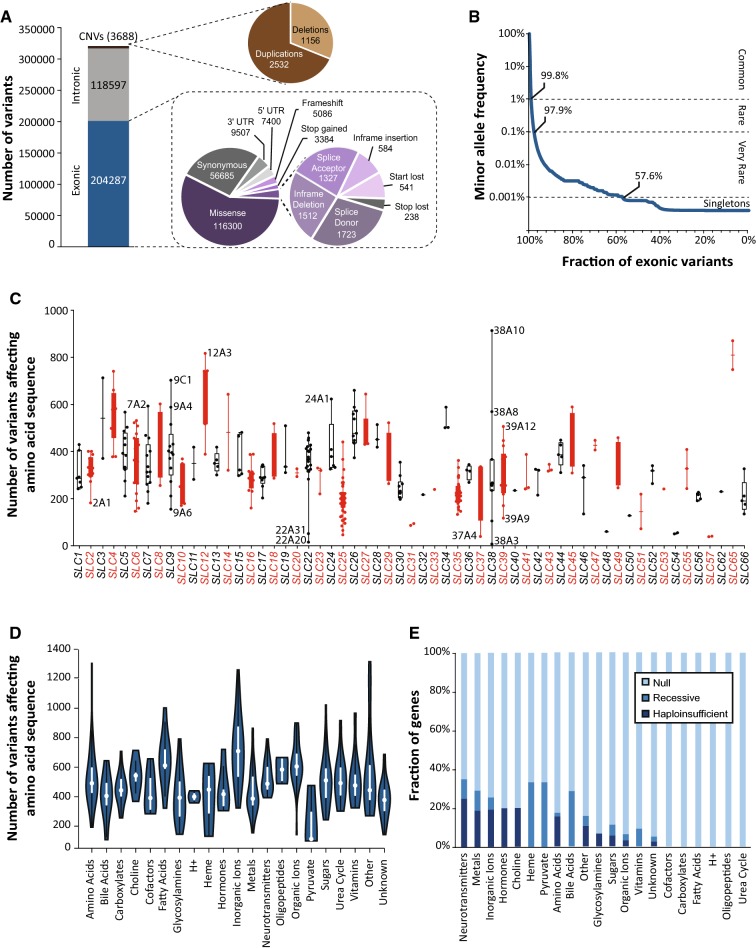
The landscape of genetic variability within the human *SLC* gene superfamily. **a** Overview of genetic variants across 401 *SLC* genes based on the Next-Generation Sequencing data of 141,456 individuals from seven major populations. Of the 204,287 identified exonic SNVs, the majority resulted in amino acid exchanges. In addition, we identified 3688 copy-number variations (CNVs) of *SLC* genes. **b** 99.8% of all exonic *SLC* variants were rare with minor allele frequencies < 1% and 57.6% were only found in a single individual. **c** Box and whisker plot depicting the number of variants that affect the amino acid sequence of the respective gene product (missense, frameshift, start-lost, stop-gain, indels, and splicing variants). Note that the number of such variants differs drastically between genes and *SLC* subfamilies. The middle line depicts the median and the edges of the boxes depict the 25th and 75th percentiles. **d** Violin plot of total exonic *SLC* variants per gene, classified by endogenous transporter substrate. White dots represent the median number of variants per gene, with the ends of the white boxes indicating the 25th and 75th percentiles. Polygons represent density estimates of the data and extend to extreme values. **e** Stacked bar plot showing the fraction of genes under high evolutionary constraint, with genes classified by protein substrate. Evolutionary constraint was estimated using the pLI score (Lek et al. 2016), with scores < 0.5 defined as little constraint (“Null”), scores 0.5 ≤ *x* ≤ 0.9 for genes for which homozygous loss-of-function results in a deleterious phenotype (“Recessive”), and scores > 0.9 defined for haploinsufficient genes

Across 141,456 unrelated individuals, we identified a total of 204,287 exonic single-nucleotide variants (SNVs) and indels (Fig. 1a). In addition, the data set contained 118,597 intronic variations; however, as these were not systematically covered, they were excluded in our further analyses. The majority of exonic variants resulted in amino acid exchanges of the encoded polypeptide (*n* = 116,300; 57% of all exonic variants). The remaining SNVs included synonymous variants (*n* = 56,685; 28%) and variants in the untranslated regions (*n* = 9507; 5% in the 5’ UTRs and *n* = 7400; 4% in the 3’ UTRs). Furthermore, we identified a multitude of variants that result in putative loss-of-function of the gene product, such as frameshifts (*n* = 5086), stop-gain variants (*n* = 3384), and variations in canonical splice sites (*n* = 3050). In addition to SNVs, we found 3688 copy-number variations (CNVs), comprised of 2532 duplications and 1156 deletions (Fig. 1a). Strikingly, of the 204,287 total exonic variants, 203,968 (99.8%) were identified as rare, with MAFs < 1% (Fig. 1b).

Next, we focused specifically on variants that affected the amino acid sequence of the encoded gene product. Among the subfamilies, variability was highest in the cholesterol transporter family *SLC65* with a median of 809 variants per gene (*n* = 2 genes), followed by *SLC12* chloride cotransporters (604 variants per gene; *n* = 9) and the bicarbonate transporter family *SLC4* (556 variants per gene; *n* = 10; Fig. 1c). By contrast, four subfamilies harbored less than 100 variants per gene (*SLC31*, *SLC48*, *SLC54,* and *SLC57*). When stratifying *SLC* genes by substrate, variability was highest in genes coding for inorganic ion transporters (718 ± 228 s.d. variants per gene) and fatty acids (646 ± 187 variants per gene), whereas pyruvate transporters (230 ± 213 variants per gene) and metal transporters (433 ± 150) harbored substantially fewer variants (Fig. 1d). Loss-of-function variants were depleted in transporters of neurotransmitters, hormones, choline, inorganic ions, and metals with 19–25% of genes being classified as haploinsufficient, suggesting high evolutionary constraints and reduced functional redundancy in these gene families (Fig. 1e). In contrast, none of the genes encoding transporters of cofactors, carboxylates, fatty acids, protons, oligopeptides, and urea cycle metabolites were found to be haploinsufficient.

### Population-specific frequencies of clinically important *SLC* variants and haplotypes

SLC transporters mediate the transport of a plethora of drugs, and multiple *SLC* variants can impact disposition, efficacy, or toxicity of various medications (Table 1 and Supplementary Table 1). Here, we analyzed the population-specific frequencies of 31 *SLC* variants with clinically relevant pharmacogenomic associations, mostly to antidiabetics, analgesics, anticoagulants, and various chemotherapeutics. Importantly, variant prevalence was highly population-specific and 42% of these variants (*n* = 13/31) differed more than fivefold between populations. Common variants in *SLC22A1* have been repeatedly linked to altered drug disposition and efficacy of metformin (Todd and Florez 2014), imatinib (Watkins et al. 2015), and various opioids (Tzvetkov 2017). Multiple variations with functional consequences, including M420del (SLC22A1*2), R61C (SLC22A1*3), G401S (SLC22A1*4), and G465R (SLC22A1*5), are absent in East Asians, whereas they can reach frequencies up to 21.9% in other populations (Table 1). In contrast, L160F, which is associated with altered imatinib pharmacokinetics and an increased risk of resistance to imatinib (Cargnin et al. 2018; Di Paolo et al. 2014; Makhtar et al. 2018), is common in East Asians (MAF = 14.2%) but lowest in Africans (3.8%).

**Table 1:** Worldwide allele frequencies of *SLC* variants associated with clinical drug response or toxicity phenotypes

Gene (protein)	Variant	Type	MAF (in %)							Association	References
			NFE	FIN	ASJ	LAT	EAS	SAS	AFR		
*SLC1A1* (EAAC1)	rs2228622:G>A	Synonymous (T138T)	41.7	33.9	51.7	40.7	24.6	33.9	21.2	Increased risk of antipsychotics-induced obsessive-compulsive symptoms (OR = 3.9)	Kwon et al. (2009)
*SLC11A1* (NRAMP1)	rs17235409:G>A	Missense (D543 N)	2.0	2.2	1.4	11.3	14.1	5.4	6.3	Increased risk of failure of tuberculosis therapy (OR = 11)	Salinas-Delgado et al. (2015)
*SLC12A3* (NCC)	rs1529927:G>C	Missense (A264G)	3.6	3.1	0.6	0.6	0.0	0.8	0.6	Increased efficacy of diuretics	Vormfelde et al. (2007)
*SLC12A6* (KCC3)	rs7164902:C>T	Synonymous (L159L)	24.3	18.1	25.1	18.3	34.2	34.0	21.6	Decreased risk of thalidomide-induced neuropathy (OR = 0.5–0.6)	Johnson et al. (2011)
*SLC15A2* (PEPT2)	rs1143671:C>T; rs1143672:G>A; rs2257212:C>T	Missense (P409S; R509 K; L350F)	45.4	40.8	37.9	24.3	71.5	29.9	48.4	Decreased warfarin dose requirement	Cai et al. (2017)
										Increased PFS of HCC patients following sorafenib therapy (OR = 0.5)	Lee et al. (2015)
*SLC16A5* (MCT6)	rs4788863:C>T	Synonymous (L41L)	26.5	18.7	31.9	38.0	65.6	25.8	25.6	Decreased risk of cisplatin-induced ototoxicity (OR = 0.06)	Drögemöller et al. (2017)
*SLC16A7* (MCT2)	rs3763980:G>T	Missense (T445S)	24.7	23.4	23.0	13.6	30.7	22.6	21.5	Increased risk of poor response to methotrexate (OR = 1.9)	Moncrieffe et al. (2010)
*SLC19A1* (RFC)	rs1051266:G>A	Missense (R27H)	43.2	45.0	39.9	43.7	52.9	40.8	61.8	Decreased risk of methotrexate-induced GI toxicity (OR = 0.38)	Lima et al. (2014)
										Increased risk of methotrexate-induced hepatotoxicity (OR = 5.3)	Suthandiram et al. (2014)
										Decreased frequency of methotrexate discontinuation due to toxicity (HR = 0.33)	Bohanec Grabar et al. (2012)
										Lower rapid response rate to irinotecan (OR = 3.6)	Huang et al. (2013)
	rs1051296:T>G	3′ UTR	42.3	43.5	38.7	40.6	54.5	46.0	45.4	Lower fraction of patients above the therapeutic threshold of methotrexate (8.6% GG vs. 40% TT; *p* = 0.02)	Wang et al. (2014)
	rs1051298:C>T	3′ UTR	43.6	45.0	38.3	45.3	54.3	N/A	51.2	Decreased survival time following pemetrexate treatment (HR = 1.8)	Corrigan et al. (2014)
*SLC22A1* (OCT1)	rs12208357:C>T	Missense (R61C)	7.7	5.6	9.5	2.3	< 0.1	2.9	1	Reduced metformin response in healthy subjects after OGTT; reduced morphine clearance and increased AUC after codeine administration; increased plasma concentrations and efficacy of tropisetron and ondansetron	Fukuda et al. (2013), Shu et al. (2007), Tzvetkov et al. (2012, 2013)
	rs35167514:ATG>del	Missense (M420del)	14.5	11.8	9.2	21.9	< 0.1	11.2	5.5		
	rs34059508:G>A	Missense (G465R)	2.3	0.7	1.3	0.7	0	0.2	0.4		
	rs34130495:G>A	Missense (G401S)	2.6	1.9	0.7	0.9	0	0.3	0.5		
	rs683369:C>G	Missense (L160F)	22.3	16.4	16.2	8.7	14.2	15.4	3.8	Decreased major molecular response to imatinib (OR = 0.6)	Cargnin et al. (2018)
										Increased risk of resistance to imatinib (OR = 1.9–3.3)	Makhtar et al. (2018)
										Increased risk of imatinib-induced conjunctival hemorrhage (OR = 4.8)	Qiu et al. (2018)
										25% decreased imatinib clearance	Di Paolo et al. (2014)
	rs628031:G>A	Missense (M408 V)	41.5	45.4	35.1	20.2	27.3	37.7	27.1	Increased risk of imatinib resistance (OR = 1.3–2.6)	Makhtar et al. (2018)
										Decreased major molecular response to imatinib (OR = 0.6)	Cargnin et al. (2018)
										Dec. risk of metformin-induced adverse GI effects (OR = 0.39)	Tarasova et al. (2012)
*SLC22A2* (OCT2)	rs316019:A>C	Missense (A270S)	10.1	6	14.7	5.2	12.8	12.5	15.2	Increased AUC of metformin	Song et al. (2008)
	rs145450955:G>A	Missense (T201 M)	0	0	0	0	0.4	0	0	Increased AUC of metformin	Song et al. (2008)
	rs316019:G>T	Missense (S270A)	10.1	6.0	14.7	5.2	12.8	12.5	15.2	Increased Cmax and AUC of metformin	Yoon et al. (2013)
										Decreased risk of cisplatin-induced ototoxicity (OR = 0.12)	Lanvers-Kaminsky et al. (2015)
*SLC22A4* (OCTN1)	rs1050152:C>T	Missense (L503F)	42.2	31	41.5	21.7	0.1	12	7.2	30% reduced renal clearance of gapapentin	Urban et al. (2008)
*SLC22A5* (OCTN2)	rs274558:A>G	Synonymous (L269L)	39.1	50.9	40.1	31.6	64.4	62.7	35.8	Increased risk of imatinib-induced edema (OR = 3.2)	Qiu et al. (2018)
*SLC22A7* (OAT2)	rs4149178:A>G	3’ UTR	15.5	15	24.5	20.4	4.3	N/A	34.6	Increased risk of anthracycline-induced cardiotoxicity	Visscher et al. (2015)
										Increased risk of severe capecitabine toxicity	Pellicer et al. (2017)
*SLC22A8* (OAT3)	rs11568482:A>T	Missense (I305F)	0.0	0.0	0.0	0.1	5.9	0.1	0.1	50% decreased renal clearance of cefotaxime	Yee et al. (2013)
*SLC22A16* (FLIPT2)	rs714368:A>G	Missense (H49R)	36.9	22.5	30.4	41.7	18.1	21.7	27.5	Increased risk of FAC-induced nausea (OR = 1.8)	Tecza et al. (2018)
										Decreased requirement for dose delay in AC therapy	Bray et al. (2010)
	rs723685:T>C	Missense (V252A)	8.8	6.8	11.7	12.1	6.9	7.5	11.2	Decreased requirement for dose delay in AC therapy	Bray et al. (2010)
	rs6907567:T>C	Synonymous (N104 N)	21.8	18.2	30.5	22.5	41.6	27.6	36.9	Increased hetatological toxicity of FAC (OR = 3.2)	Tecza et al. (2018)
										Decreased requirement for dose delay in AC therapy	Bray et al. (2010)
	rs12210538:T>C	Missense (M409T)	23.5	15.8	27.4	11.7	< 0.1	11.3	3.8	Increased requirement for dose delay in AC therapy	Bray et al. (2010)
*SLC28A1* (CNT1)	rs2242046:G>A	Missense (D521 N)	50.1	45.2	55.3	20.8	7.6	30.3	8.8	Increased risk of gemcitabine-induced hematologic toxicity	Soo et al. (2009)
*SLC28A2* (CNT2)	rs1060896:C>A	Missense (S75R)	65.4	59.2	58.3	30.2	8.6	47.8	17.6	Increased OS of NSCLC patients on gemcitabine	Soo et al. (2009)
	rs11854484:C>T	Missense (P22L)	62.2	58.2	50.9	28.4	7.9	45.4	17.1	Increased risk of gemcitabine-induced hematologic toxicity but increased OS of NSCLC patients	Soo et al. (2009)
										Increased ribavirin serum levels	Rau et al. (2013)
										Increased risk of anemia in HCV patients treated with telaprevir or boceprevir (OR = 2.3)	Ampuero et al. (2015)
*SLC28A3* (CNT3)	rs56350726:A>T	Missense (Y513F)	5.9	12.7	4.9	2.8	11.5	7.6	15.4	Higher chance of reaching sustained virological response in HCV treatment	Rau et al. (2013)
										twofold reduced incidence of anemia in HCV patient treated with ribavirin	Doehring et al. (2011)
*SLC30A8* (ZnT8)	rs16889462:G>A	Missense (R325Q)	0.1	0.0	0.2	0.6	8.0	0.3	10.5	Increased repaglinide efficacy	Huang et al. (2010)
	rs13266634:C>T	Missense (R325 W)	30.4	38.0	26.1	25.7	44.0	23.0	8.9	Increased repaglinide efficacy	Huang et al. (2010)
*SLC30A9* (ZnT9)	rs1047626:G>A	Missense (M50 V)	23.8	20.3	29.1	19.3	4.1	22.4	80.2	Decreased risk of aspirin-exacerbated respiratory disease (OR = 0.13)	Shin et al. (2014)
*SLC47A1* (MATE1)	rs2252281:T>C	5′ UTR	31.2	23.3	36.0	17.6	21.5	N/A	35.1	Enhanced metformin response	Stocker et al. (2013)
*SLC47A2* (MATE2-K)	rs12943590:G>A	5′UTR	27.2	26.7	34.8	34.5	46.9	N/A	21.5	Decreased metformin response	Choi et al. (2011) and Stocker et al. (2013)

*MAF* minor allele frequency, *NFE* non-Finnish Europeans, *FIN* Finns, *ASJ* Ashkenazi Jews, *LAT* Latinos, *EAS* East Asians, *SAS* South Asians, *AFR* Africans, *Cmax* peak serum concentration, *AUC* area under the concentration time curve; *HCC* hepatocellular carcinoma; *NSCLC* non-small cell lung cancer; *OR* odds ratio; *HR* hazard ratio; *PFS* progression-free survival; *GI* gastrointestinal; *FAC* fluorouracil, anthracycline and cyclophosphamide combination therapy; *AC* anthracycline and cyclophosphamide combination therapy; *OS* overall survival; *OGTT* oral glucose tolerance test

While pharmacogenetically important polymorphisms in the nucleoside transporter genes *SLC28A1* and *SLC28A2* were common worldwide, their frequencies differed drastically between populations. Rs2242046 in *SLC28A1*, as well as the highly linked variants rs1060896 and rs11854484 in *SLC28A2* (*R*^2^ = 0.9), were least common in East Asian and African populations (MAF = 7.6–17.6%), whereas they were consistently more prevalent in all other populations (MAF = 20.8–65.4%). In contrast, Africans and East Asians were among the populations with the highest frequency of rs56350726, a variant in *SLC28A3* implicated in improved outcomes and reduced toxicity of antiviral hepatitis C virus (HCV) therapy (Doehring et al. 2011; Rau et al. 2013), suggesting potentially important implications for toxicity risk of nucleoside analogs used in the treatment of viral infections and various cancers. Pronounced tenfold differences between populations were furthermore observed for the missense variant rs17235409 in *SLC11A1* that is implicated in treatment failure of patients with pulmonary tuberculosis to isoniazid, rifampicin, pyrazinamide, and ethambutol combination therapy (Salinas-Delgado et al. 2015).

The variant rs1529927 in the renal sodium and chloride reabsorption transporter *SLC12A3* (NCC) is associated with increased efficacy of diuretics (Vormfelde et al. 2007) and was common in Europeans (MAF = 3.1–3.6%), but rare in all other populations studied. Similar population specificity was observed for the reduced function variant rs11568482 in *SLC22A8* (OAT3), which was exclusively found in East Asians with frequencies of 5.9%, with important implications for the renal clearance of the OAT3 substrate cefotaxime (Yee et al. 2013). Moreover, rs16889462 in *SLC30A8*, a variant associated with increased response to repaglinide (Huang et al. 2010), was common in Africans (MAF = 10.5%) and East Asians (MAF = 8%), but rare in all other populations analyzed (MAF < 1%).

### Genetic variants in human *SLC* genes are predicted to substantially contribute to inter-individual differences in transporter function

**Fig. 2:**
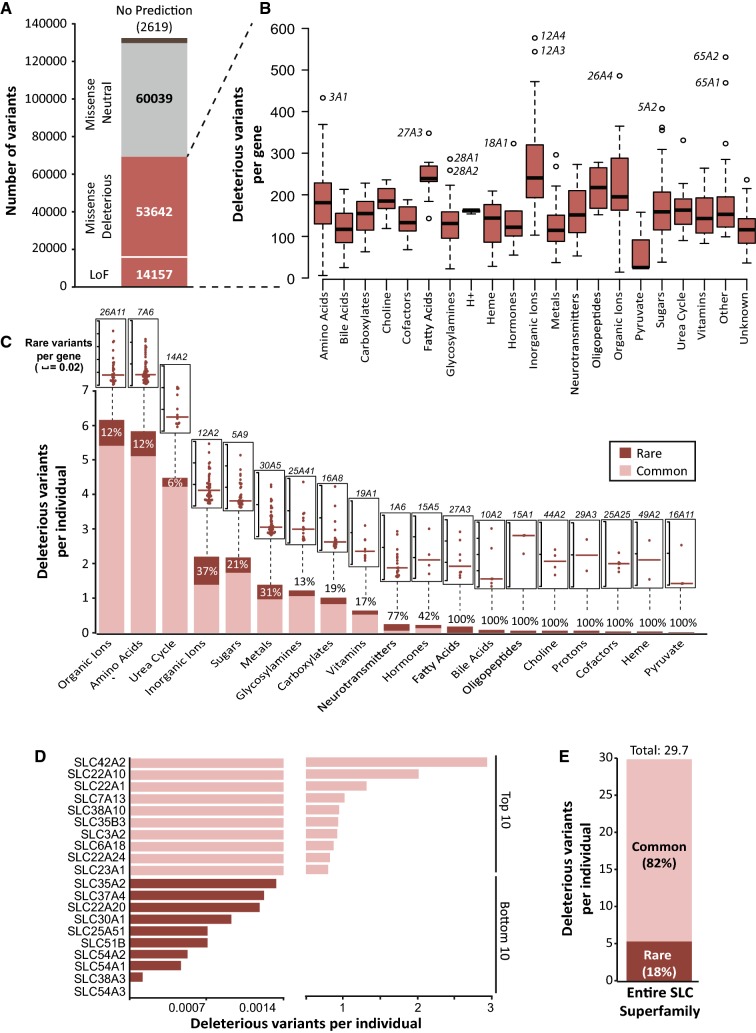
Rare genetic variants contribute considerably to the genetically encoded functional variability of SLC transporters. **a** Of the 116,300 identified missense variants, 53,642 were predicted to alter the functionality of the transporter protein. Furthermore, 14,157 variants that caused frameshifts, the loss of a start or the premature gain of a stop codon, or variants that affecting canonical splice sites were expected to result in a loss of protein function. **b** Box and whisker plot of all these deleterious variants (*n* = 67,799) per gene demonstrates that the complexity of genetically encoded functional variability differs drastically between SLC substrate classes. **c** When aggregating variant numbers per individual, most variants were identified in transporters of organic ions and amino acids. Common variants (MAF > 1%) are shown in light red, while rare variants (MAF < 1%) are shown in dark red. Percentage values within or above stacked columns indicate the fraction of the genetically encoded functional variability allotted to rare variants. Inlet dot plots depict the total rare deleterious *SLC* variants per individual per gene, with the median represented by the dark bar. The gene with the highest number of rare deleterious variants per substrate class is indicated above the inlet. **d** The aggregated frequency of variants that affect transporter function is plotted for the top 10 and bottom 10 *SLC* genes. Note that differences between the most and least variable genes exceed 1000-fold. **e** Across the entire SLC superfamily, each individual was found to harbor 29.7 variants that are predicted to affect the functionality of the encoded transporter protein. Of this genetically encoded functional variability, 18.7% is attributed to rare variants

To gauge the functional effects arising from the observed genetic variability beyond variants with known pharmacogenetic associations, we employed an array of 13 partly orthogonal computational algorithms. Of the 116,300 identified missense variants, 53,642 (46%) were predicted to alter the functionality of the respective gene product (Fig. 2a). Furthermore, we considered all 14,157 variants that caused frameshifts, the loss of a start or the premature gain of a stop codon or variants affecting canonical splice sites as putative loss-of-function variants. The highest median numbers of functional variants were identified in transporters of inorganic ions (239 variants), fatty acids (238 variants), and oligopeptides (216 variants; Fig. 2b). Per gene, most putatively deleterious variants were found in *SLC12A4* (577 variants), *SLC12A3* (544 variants), and *SLC65A2* (531 variants), whereas less than 25 variants were found in pyruvate transporters.

Each individual was found to harbor on average 6.2 and 5.8 variants with functional effects in organic ion and amino acid transporters, respectively (Fig. 2c). In contrast, the average diploid human genome contained less than 0.1 variants in transporters of bile acids, oligopeptides, choline, protons, heme, pyruvate, and various cofactors. The contribution of rare variants differed considerably between substrate classes. While rare variants accounted for 6%, 12%, and 12% of the genetically encoded functional variability in urea cycle, organic ion, and amino acid transporters, no common variants with functional effects were identified in transporters of pyruvate, heme, or various other substrates and, thus, rare genetic variants were the only cause of genetically encoded functional effects in these transporters (Fig. 2c). *SLC26A11*, *SLC10A2,* and *SLC26A10* harbored most rare functional variants per individual, whereas least were found in *SLC54A1*, *SLC54A2,* and *SLC51B*. When integrating rare and common variant data, most deleterious variants were identified in the putative ammonium transporter *SLC42A2* (RhBG; 3 deleterious variants per individual), the poorly understood ion transporter *SLC22A10* (OAT5; 2 variants per individual), and the highly clinically relevant drug transporter *SLC22A1* (OCT1; 1.3 variants per individual; Fig. 2d). In contrast, less than 1 in 2000 individuals harbored a deleterious variant in *SLC54A3* (0), *SLC38A3* (0.0001), *SLC54A1* (0.0005), *SLC51B* (0.0007), *SLC25A51* (0.0007), and *SLC30A1* (0.0009). Strikingly, when aggregating information about genetically encoded functional variability across the entire *SLC* superfamily of genes, each individual was found to harbor on average 29.7 variants with putative functional consequences in *SLC* transporters of which rare variants accounted for 18% (5.4 rare variants per individual; Fig. 2e).

### Genetic variability in *SLC* genes is highly population specific with important consequences for the predisposition to Mendelian disease

**Fig. 3:**
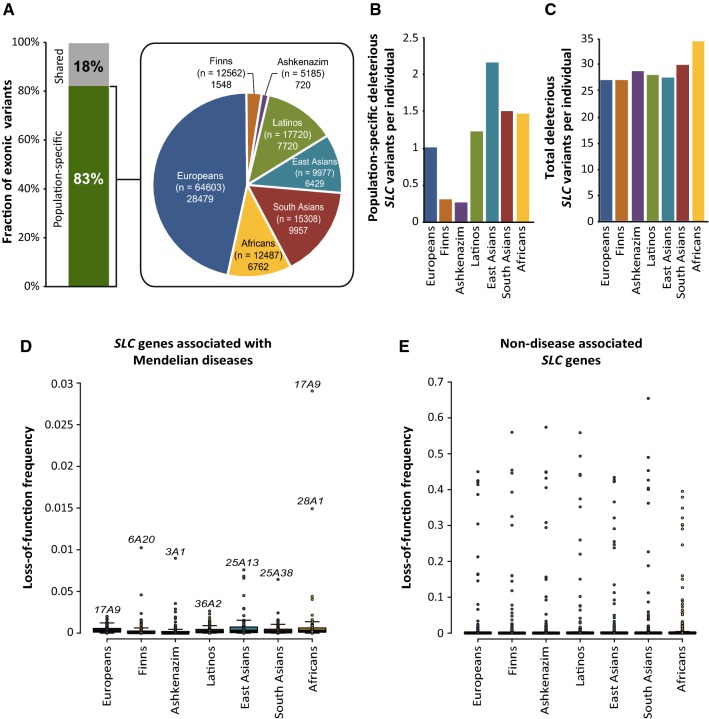
Genetic *SLC* transporter variability is highly population-specific. **a** Of all putatively deleterious variants (*n* = 67,799), 83% were only detected in a single population. The pie chart depicts the total number of deleterious, population-specific variants for each population. Values in brackets indicate the size of the cohort for the population in question. **b** The number of population-specific, deleterious *SLC* variants per individual differed considerably across major human populations. **c** In contrast, only minor differences in overall genetically encoded functional *SLC* variability per individual were observed across populations. **d**, **e** Dot plots depicting the cumulative frequency of putative loss-of-function (LoF) variants (frameshifts, start-lost, and stop-gain variations, as well as variants affecting canonical splice sites) per gene for *SLC* genes associated with Mendelian diseases **(d)** as well as for non-disease-associated genes **(e)**. Note that LoF frequency of disease-associated genes is much lower than of non-disease-associated genes

When we stratified the identified *SLC* variants that were predicted to affect transporter function by ancestry, we found that the distribution varied drastically between populations, with 83% of variants (*n* = 56,273) restricted to a single population (Fig. 3a). Most population-specific variants were identified in Europeans, whereas the lowest numbers were found in Finns and Ashkenazi Jews, at least in part due to unequal cohort sizes. Interestingly, after adjusting for cohort size, we found that East Asians had the largest number of population-specific variants with predicted functional consequence, suggesting that this population might benefit most from population-adjusted genotyping strategies (Fig. 3b). In contrast, overall genetically encoded functional variability differed only moderately between populations with individuals of African (34.6 variants/ individual) and European (28.6 variants/ individual) ancestry carrying on average the most and least deleterious variants, respectively (Fig. 3c).

Next, we focused specifically on genes for which loss-of-function mutations are associated with Mendelian disorders. Notably, the cohorts which we analyzed were sampled from the general population and individuals with severe congenital diseases were excluded. However, we hypothesized that quantification of loss-of-function carrier frequencies in the general population could be used as a proxy for Mendelian disease risk with recessive mode of inheritance. To this end, we aggregated frequencies of frameshifts and stop-gain variations, as well as variants affecting canonical splice sites removed variants previously reported to not cause disease (see “Materials and methods”). Overall, 109 out of 401 human *SLC* genes were found to have known associations with genetic diseases, of which 84 were autosomal recessive (Supplementary Table 2).

As expected, the frequency of loss-of-function variants was substantially lower in disease-associated *SLC* genes compared to *SLC* genes that were not associated with genetic disease (Fig. 3d, e). To evaluate whether this approach was indeed suitable to identify population-specific disease risk, we focused on Mendelian disorders with well-established ethnogeographic variation. Loss-of-function of the amino acid transporter SLC3A1 has been associated with cystinuria (OMIM 220100). The disease has a worldwide prevalence of around 1 in 7000 neonates and has been reported to be most common among Jews with frequencies up to 1:2500 individuals in certain subpopulations (Eggermann et al. 2012). Interestingly, we found highest loss-of-function frequencies of *SLC3A1* in Ashkenazi Jews (0.9%), in agreement with previous reports (Pras et al. 1995). Accordingly, one in 12,345 Ashkenazim individuals can be expected to be homozygous for an *SLC3A1* loss-of-function variant. Carrier rates in other populations were > tenfold lower. Similarly, variability profiles of the aspartate transporter *SLC25A13* recapitulated increased prevalence of type II citrullinemia (OMIM 605814) in East Asians (Lu et al. 2005), with aggregated loss-of-function frequencies of 0.8%, corresponding to 1 in 15,625 homozygous East Asian carriers.

Lysinuric protein intolerance (OMIM 222700) is most prevalent in the Finnish population and has been associated with mutations in *SLC7A7* (Torrents et al. 1999). Importantly, loss-of-function frequencies of *SLC7A7* in Finns were more than fivefold higher than in other populations. Similarly, our data aligned with reported population differences in the genetic basis of Pendred syndrome (OMIM 274600), the most common form of syndromic genetic deafness. While genetic variation in *SLC26A4* is a major cause of these disorders in Asia, mutations in different genes have been reported to be the most important factors in Western populations (Park et al. 2003). In agreement with these genetic roots, frequencies of *SLC26A4* loss-of-function variants in East Asian populations were approximately sixfold higher than in Europeans. Based on the results, we conclude that the analysis of loss-of-function frequencies in the general population can be a powerful resource to inform about disease risk and population-specific genetic complexity underlying recessive Mendelian diseases.

### Structural consequences of *SLC* variability

To obtain mechanistic insights into the effects of SLC variability, we mapped the genetic variants to the corresponding 3D structures of the transporter proteins. To this end, we focused on transporters with important roles in human physiology and pharmacology for which high-resolution crystal structures were either available or could be modeled with high confidence.

The glucose transporter GLUT1 encoded by *SLC2A1* facilitates glucose uptake into erythrocytes and is the major glucose transporter in the human blood–brain barrier. Variations in GLUT1 can cause GLUT1 deficiency syndrome with an autosomal dominant inheritance pattern, which presents as neurological problems, developmental delays, complex movement disorders, and, occasionally, hemolytic anemia (De Giorgis and Veggiotti 2013). GLUT1 belongs to the major facilitator superfamily (MFS) of transporters and consists of two discretely folded domains, termed N- and C-domain, each consisting of six transmembrane helices, that are connected by an intracellular helical bundle (ICH) (Deng et al. 2014). To translocate glucose, GLUT1 undergoes structural changes and alternates between inwards and outwards facing confirmations and the ICH has been shown to play essential roles in this process (Yan 2013). The ICH interacts with multiple transmembrane domains (TMDs) of GLUT1, thereby acting as a latch that, in the absence of a ligand, stabilizes GLUT1 in the outward facing confirmation (Deng et al. 2014). Upon ligand binding, interactions between the N and C domains are altered, resulting in a transition towards the inward-open state.

**Fig. 4:**
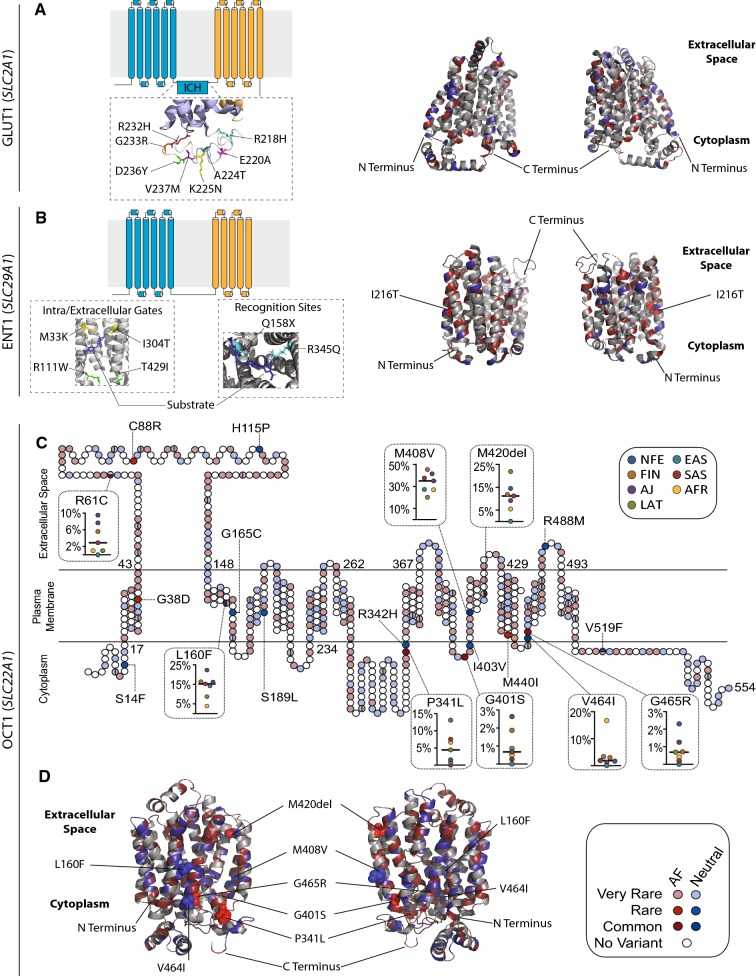
Structural mapping of GLUT1, ENT1, and OCT1 variability. Schematic topology models and experimentally derived 3D protein structures viewed from both sides of the membrane plane are shown for human GLUT1 (**a**) and ENT1 (**b**). Detailed 3D structures of key protein domains with functionally relevant variants (sticks) and substrates (sticks in dark blue) are shown as insets under the respective topology models. Red segments of the 3D models represent residues with putatively deleterious variants, blue segments represent residues with putatively neutral variants, and gray segments represent residues for which no associated variants have been identified in 141,456 individuals. Deleterious and neutral common variants are depicted as red and blue spheres, respectively. ICH = intracellular helical bundle; **c** schematic representation of the secondary structure of human OCT1, with all deleterious (shades of red) and neutral (shades of blue) variants mapped to their respective residues. Color intensity of each residue indicates variant frequencies. Population-specific frequencies of the common variants R61C, L160F, P341L, G401, M408 V, M420del, V464I, and G465R are shown for Africans (AFR; yellow), Ashkenazi Jews (AJ; purple), Non-Finnish Europeans (NFE; dark blue), Latinos (LAT; green), East Asians (EAS; light blue), and South Asians (SAS; red) in inlets. **d** The predicted 3D model of OCT1 viewed from both sides of the membrane plane is shown. Red segments of the 3D models represent residues with putatively deleterious variants, blue segments represent residues with putatively neutral variants, and gray segments represent residues for which no associated variants have been identified in 141,456 individuals. Deleterious and neutral common variants are depicted as red and blue spheres, respectively. Note that R61C is not depicted, because the corresponding fold could not be accurately modeled

In total, 181 variants in GLUT1 were identified that were distributed across all domains of the protein, including the ICH (Fig. 4a). Notably, we identified rare variations in R400, which participates in stabilization of the interaction between the N- and C-terminal domains (Park 2015), as well as in R92, R93, R232, and E209, which form a tightly connected salt bridge network that controls GLUT1 state transitions (Galochkina et al. 2019). In contrast, no variants were observed in the glucose entry pocket (N34, V69, R126, and Y292) or in the amino acids lining the central glucose cavity (S73, Q279, Q282, Q283, N288, N411, and N415). As the analyzed cohort was depleted of patients with congenital diseases, these findings suggest that GLUT1 function can be permissive to variations that modulate salt bridges involved in state transitions, whereas residues directly involved in glucose translocation appear more conserved.

ENT1 encoded by *SLC29A1* is an essential uptake transporter of nucleosides and nucleoside analogs. As such, ENT1 is of tremendous pharmacological importance for the disposition of various antiviral and antineoplastic medications, and is itself the pharmacological target of multiple antiarrhythmic and antihypertensive medications (Boswell-Casteel and Hays 2017). The organization in N- and C-terminal pseudo-symmetric domains as well as the transport cycle of state transitions are overall similar between ENT1 and GLUT1. However, the C-domain of ENT1 only comprises five instead of six TMDs (Wright and Lee 2019). In total, our data set contained 263 variants that affected the ENT1 amino acid sequence. The only common *SLC29A1* variant was rs45573936, with a frequency of 1.8%, resulting in an I216T amino acid substitution in TMD6 that alters binding affinity of the adenosine analog inhibitor NBMPR and has been implicated in neurological symptoms upon alcohol withdrawal (Kim et al. 2011). We identified rare variants in multiple critical residues of the central cavity of the transporter that likely impact substrate affinity and binding kinetics. These include a substitution of the hydrophobic methionine M33 that lines the narrowest constriction point at the extracellular side (Wright and Lee 2019) with a charged lysine, as well as amino acid exchanges affecting various residues that define the intracellular gate, such as R111, I304, and T429 (Fig. 4b). Furthermore, multiple variants affected the residues M89, Q158, and R345 that have been shown to directly interact with the structurally heterogeneous ENT1 inhibitors dilazep and NBMPR (Wright and Lee 2019). We conclude that the genetic variability in *SLC29A1* is extensive and multiple variants are highly likely to affect ENT1 pharmacology.

In contrast to GLUT1 and ENT1, no crystal structure of human OCT1 has yet been presented. However, structure–function relationships have been inferred from crystal structures of homologous fungal transporters (Pedersen et al. 2013) and mutagenesis studies using rat OCT1 (Gorbunov et al. 2008; Popp et al. 2005). Here, we used the computational tool Phyre2 (Kelley et al. 2015) to predict the structure of human OCT1 based on multiple sequence alignments and homologous experimentally determined models. We could derive a high confidence model (100% confidence score) covering 82% of the human OCT1 protein sequence, which aligned well with the putative structure of rat OCT1 (Supplementary Fig. 1).

*SLC22A1* harbors eight common variants that affect OCT1 amino acid sequence, of which R61C, G401S, and G465R resulted in strongly reduced OCT1 function in vitro, whereas substrate-specific results have been reported for M420del and P341L (Choi and Song 2012; Shu et al. 2003; Tzvetkov et al. 2011, 2012). The function of OCT1 isoforms carrying V464I, L160F or M408 V was not found to be altered in vitro (Shu et al. 2003). Notably, M420del occurs exclusively together with M408 V, whereas M408 V can occur in isolation (*D*′ = 1, *R*^2^ = 0.061) (Tzvetkov et al. 2014). The frequencies of these common variants varied greatly across populations (Fig. 4c) with MAFs of the M240del variant ranging from 0.09% in East Asians to 21.9% in Latinos and M408 V from 20.2% in Latinos to 45.4% in Finns in accordance with previous reports on smaller cohorts (Seitz et al. 2015). In total, we identified nine OCT1 variants with a global MAF > 0.1%, half of which were localized to TMDs (G38D, M420del, M440I, and G465R localized to TM1, TM9, TM10, and TM11, respectively) (Fig. 4c). In addition to these well-characterized variants, we found 445 additional variants that alter OCT1 amino acid sequence (Fig. 4c, d). Rare variations were found to affect the mechanistically important residues S358, R439, I446, Q447, and C450, which are directly involved in the coordination of cationic substrates, as well as F485, a residue essential for state transition during substrate translocation (Gorbunov et al. 2008; Pedersen et al. 2013; Volk et al. 2009). Combined, structural mapping of the genetic variability in physiologically and pharmacologically important SLC transporters supports the conclusion that rare, as of yet uncharacterized variants, are likely to have important functional impacts on transporter structure and function.

## Discussion

SLC transporters play pivotal roles in diverse physiological processes, including the uptake and disposition of nutrients, maintenance of acid-base homeostasis, neurotransmission, and the elimination of metabolic products. In addition, they are involved in the disposition of a multitude of clinically relevant medications, ranging from chemotherapeutics to antidiuretics. Their biological importance and pharmacological relevance, as well as their roles in numerous human diseases, render SLC transporters attractive drug targets. Current clinical applications include the targeted treatment of hypertension (inhibition of *SLC12A1*/NKCC2 by diuretics), diabetes (inhibition of *SLC5A2*/SGLT2 by gliflozins), gout (inhibition of *SLC22A12*/URAT1 by lesinurad), schizophrenia (inhibition of *SLC6A9*/GLYT1 by bitopertin), and depression (inhibition of *SLC6* transporters by serotonin-selective reuptake inhibitors) (Lin et al. 2015). Our analyses revealed that almost half of all *SLC* alleles associated with altered drug response or toxicity had frequencies that differed more than fivefold between populations. These findings have important implications for the treatment with SLC transporter substrates in an ethnogeographic context, and incentivize the adoption of population-adjusted genotyping strategies to optimize patient outcomes.

In addition to the previously described *SLC* alleles, we identified a surprising extent of genetic complexity within *SLC* transporters. Importantly, less than 0.2% of all identified variants were found in more than 1% of alleles and more than half of all variants were singletons. To estimate the overall contribution of this plethora of rare variants to functional SLC variability, we used an array of 13 partly orthogonal computational algorithms that leverage sequence information, evolutionary conservation, structural considerations, and functional genomics data in the prediction process, and have been found to perform reasonably well on both disease-associated and pharmacogenomic data sets (Li et al. 2018; Zhou et al. 2019). These analyses revealed that each individual genome harbors on average 29.7 putatively functional *SLC* variants, with rare variants accounting for 18% of this genetically encoded functional variability. Notably, nearly half of all putatively deleterious *SLC* variants in an individual affected transporters of amino acids, organic ions, and urea cycle intermediates. Structural mapping of the portfolio of genetic variants on available crystal structures of the encoded proteins revealed that rare variants affect multiple residues that have been shown to be essential for transporter function, thus further corroborating the important functional roles of rare genetic variability. Besides variations that are directly involved in substrate coordination or translocation, a variety of missense variants in SLC transporters are known to affect transporter function by altering subcellular trafficking or localization. Prominent examples include variants in SLC22A1 (Chen et al. 2010), SLC12A6 (Salin-Cantegrel et al. 2011) and SLC30A5 (Thornton et al. 2011). Notably, while these effects are difficult to infer by structural mapping, variant effect predictors, such as those used in this study, faithfully predicted localization defects and even outperformed specialized subcellular localization tools (Orioli and Vihinen 2019).

Strikingly, we found that 83% of all variants that were predicted to affect SLC function were population-specific. This degree of inter-ethnic variability is similar to other highly variable pharmacogene families, such as *CYPs* (Fujikura et al. 2015) and *UGTs* (Kaniwa et al. 2005), as well as to the related *SLCO* family of transporters (Zhang and Lauschke 2019). While individuals of African ancestry harbored most functional *SLC* variants in agreement with previous findings of greater levels of genetic diversity in Africans compared to non-African populations (Campbell and Tishkoff 2008; Tishkoff et al. 2009), the largest number of population-specific *SLC* variants was identified in East Asians. Interestingly, when focusing on *SLC* genes associated with Mendelian disease, we found that population-specific carrier frequency in the general population recapitulated the ethnogeographic variation of various Mendelian disorders with a recessive mode of inheritance, including cystinuria in Jewish individuals, type II citrullinemia in East Asians, and lysinuric protein intolerance in Finns. We thus conclude, in agreement with previous studies (Fujikura 2016), that NGS data of the general population can provide a suitable tool for the analysis of the genetic variability underlying inherited disorders. Furthermore, we argue that the presented data can serve as a unique large-scale resource for clinical geneticists to inform about population-specific prevalence and allelic composition of risk alleles associated with Mendelian diseases of SLC transporters. Importantly, the approach is likely not suitable for the analyses of diseases following a dominant mode of inheritance, as individuals with severe congenital diseases were excluded from the analyzed cohorts, resulting in an underestimation of dominant disease allele frequencies in our data set. Notably, the relatively high frequencies of loss-of-function variants in *SLC28A1*, which are associated with autosomal dominant uridine-cytidineuria (OMIM 618477), might be explained by its putatively benign nature (Wevers et al. 2019).

NGS is already widely and successfully applied in the diagnosis of rare monogenic diseases (Boycott et al. 2013; Fernandez-Marmiesse et al. 2018). However, while targeted sequencing panels that include multiple *SLC* transporters have been developed (Gordon et al. 2016; Gulilat et al. 2019; Klein et al. 2019), the incorporation of these genetic data into personalized pharmacogenomic recommendations and clinical decision-making is lagging behind. In the absence of feasible experimental strategies to characterize the functional impact of the plethora of rare *SLC* variants, computational methods have to be used. While such in silico interpretations of pharmacogenetic variants do not yet have sufficient accuracy to warrant direct clinical implementation (Zhou et al. 2018), these tools can already be used to flag patients with suspicious variants in key pharmacogenes for closer monitoring to anticipate detrimental drug response as early as possible. However, whether NGS coupled with computational pharmacogenomic analyses can indeed facilitate informed decision-making and provide a cost-effective measure to improve patient care, remains to be evaluated in prospective trials (Lauschke and Ingelman-Sundberg 2016, 2018).

In summary, by leveraging consolidated NGS data from 141,456 individuals, we comprehensively assessed the genetic variability of the human *SLC* transporter superfamily on an unprecedented scale. We demonstrate that *SLC* genes are highly variable and each individual genome is estimated to contain around 30 variants that affect SLC transporter function. The vast majority of variants were rare, and computational analyses based on evolutionary, structural, and functional genomics data indicate that these rare variants contribute approximately 20% to the genetically encoded functional variability of SLC transporters. Thus, these data serve as a powerful resource for the worldwide pattern of *SLC* variability and motivate the integration of comprehensive NGS-based genotyping into personalized predictions of SLC substrate disposition and precision public health.

## Electronic supplementary material

Below is the link to the electronic supplementary material.

**Supplementary Table** **1.** Overview of the 401 *SLC* genes included in the dataset, including their respective endogenous and drug substrates. Data obtained from http://slc.bioparadigms.org (XLSX 68 kb)

**Supplementary Table** **2.***SLC* genes and their known Mendelian disease associations, including OMIM identification numbers and mode of inheritance are shown. The cumulative frequency of putative loss-of-function variants (frameshifts, stop-gain and canonical splice site variants) is shown for each gene. AR = autosomal recessive; AD = autosomal dominant; XL = X-linked; XLR = X-linked recessive, XLD = X-linked dominant; U = unknown (XLSX 115 kb)

**Supplementary Fig.** **1.** Comparison of the predicted tertiary structures of human (blue) and rat OCT1 (red) (PDF 6128 kb)
