# Supplementary figures and images for: The genetic landscape of the human solute carrier (SLC) transporter superfamily
Source: Hum Genet. 2019 Nov 2;138(11):1359–77. doi: 10.1007/s00439-019-02081-x (PMC6874521; doi:10.1007/s00439-019-02081-x)

# Supplementary Figure 1

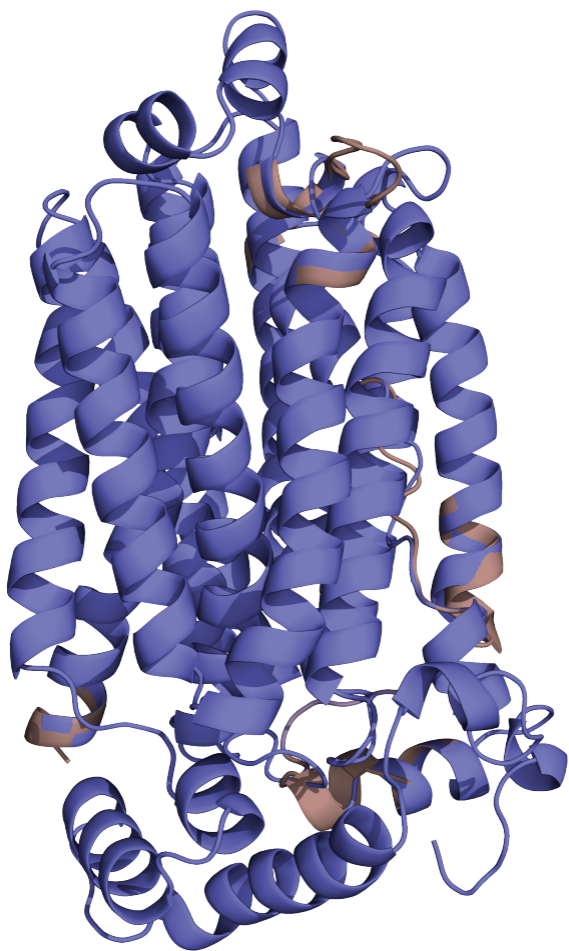

Supplement: Supplementary file 3 — Supplementary Fig. 1. Comparison of the predicted tertiary structures of human (blue) and rat OCT1 (red) (PDF 6128 kb) [file 439_2019_2081_MOESM3_ESM.pdf]
